# Evaluation of Positive Choices, a National Initiative to Disseminate Evidence-Based Alcohol and Other Drug Prevention Strategies: Web-Based Survey Study

**DOI:** 10.2196/34721

**Published:** 2022-08-26

**Authors:** Lexine Ann Stapinski, Smriti Nepal, Tara Guckel, Lucinda Rachel Grummitt, Cath Chapman, Samantha Jane Lynch, Siobhan Maree Lawler, Maree Teesson, Nicola Clare Newton

**Affiliations:** 1 The Matilda Centre for Research in Mental Health and Substance Use The University of Sydney Camperdown Australia; 2 SAX Institute Glebe Australia

**Keywords:** alcohol and other drugs, prevention, adolescence, schools, drug prevention, drug prevention website

## Abstract

**Background:**

To prevent adolescents from initiating alcohol and other drug use and reduce the associated harms, effective strategies need to be implemented. Despite their availability, effective school-based programs and evidence-informed parental guidelines are not consistently implemented. The *Positive Choices* alcohol and other drug prevention initiative and website was launched to address this research and practice gap. The intended end users were school staff, parents, and school students. An 8-month postlaunch evaluation of the website showed that end users generally had positive feedback on the website’s usability, and following its use, most of them would consider the evidence base and effectiveness of drug education resources. This study extends this initial evaluation by examining the effectiveness and impact of the *Positive Choices* initiative over a 3-year period.

**Objective:**

Guided by the five dimensions of the RE-AIM (reach, effectiveness, adoption, implementation, and maintenance) framework, the study assessed the impact of the *Positive Choices* initiative in increasing awareness and implementation of evidence-based drug prevention.

**Methods:**

Data were collected between 2017 and 2019, using web-based evaluation and community awareness surveys. Data from the surveys were merged to examine reach, effectiveness, adoption, implementation, and maintenance using descriptive statistics. Google Analytics was used to further understand the reach of the website. The System Usability Scale was used to measure website usability. In addition, inductive analysis was used to assess the participants’ feedback about *Positive Choices*.

**Results:**

A total of 5 years after launching, the *Positive Choices* website has reached 1.7 million users. A national Australian campaign increased awareness from 8% to 14% among school staff and from 15% to 22% among parents after the campaign. Following a brief interaction with the website, most participants, who were not already following the recommended strategies, reported an intention to shift toward evidence-based practices. The System Usability Scale score for the website was *good* for both user groups. The participants intended to maintain their use of the *Positive Choices* website in the future. Both user groups reported high level of confidence in communicating about topics related to alcohol and other drugs. Participants’ suggestions for improvement informed a recent website update.

**Conclusions:**

The *Positive Choices* website has the capacity to be an effective strategy for disseminating evidence-based drug prevention information and resources widely. The findings highlight the importance of investing in ongoing maintenance and promotion to enhance awareness of health websites. With the increased use and acceptability of health education websites, teams should ensure that websites are easy to navigate, are engaging, use simple language, contain evidence-informed resources, and are supported by ongoing promotional activities.

## Introduction

### Background

Adolescence is marked by considerable emotional, social, and physical changes, including increasing autonomy from parents, greater influence from peers, and increased likelihood to engage in risk-taking behaviors [1]. Corresponding to this period of experimentation, harms associated with risky behaviors such as alcohol and other drug (AOD) use peak during adolescence and early adulthood [2-4]. Globally, alcohol, tobacco, and cannabis are the most commonly used drugs among adolescents [4]. This also holds true for Australia, where a 2017 national survey among secondary school students aged 12 to 17 years showed that 46% had consumed a full serve of alcohol, 14% had used cannabis, and 13% had smoked cigarettes in the past year [5]. Early initiation of AOD use is associated with a range of negative outcomes including poor school performance, truancy, school dropout, juvenile offending, and increased risk of drug dependence and mental illness during adulthood [6-9]. To interrupt this trajectory and reduce the harms associated with AOD use, effective strategies are needed to prevent the onset and escalation of their use.

Studies have identified a number of modifiable individual risk factors that are consistently associated with AOD use among adolescents [10]. They include peer AOD use, low self-efficacy to refuse alcohol or other drugs, poor school engagement and connectedness, and mental health disorders such as depression and attention-deficit/hyperactivity disorder [10-12]. Studies also highlight the importance of parents in influencing and preventing adolescents’ AOD use. Recent evidence shows an association between parental supply of alcohol and increased risk of alcohol-related harm during adolescence and early adulthood [13,14]. This has challenged the commonly held perception that allowing teenagers to drink alcohol under parental supervision protects them against alcohol-related harms. Similarly, less restrictive parental attitudes toward alcohol use are likely to lead to earlier and more frequent alcohol use and increased drunkenness among adolescents [15].

Prevention programs targeting individual and parental risk factors are effective in reducing AOD use among adolescents [16-22]. A number of prevention programs implemented during secondary school have consistently demonstrated effectiveness in reducing AOD use [18,21], and school-based delivery offers a number of advantages including tailoring of messages to students’ developmental level and universal delivery to achieve wide reach [22,23]. Internet- and computer-based AOD prevention programs offer additional benefits in terms of engaging youth and increasing implementation fidelity and have been found to be effective in reducing adolescent AOD use [17]. Despite the growing evidence base, only a small proportion of schools implement effective AOD prevention strategies. A 2003 review of 3 decades of AOD education studies concluded that worldwide, effective AOD prevention is not widely implemented [24]. More recently, our 2017 survey of Australian schoolteachers found that <25% of teachers had implemented evidence-based AOD education programs. The study identified lack of confidence, resources, time, and support from school; attitude of parents and students; and difficulty in communicating as the main barriers to implementing programs [25].

Parenting strategies that are consistently associated with delayed initiation of alcohol use include parental monitoring, limited access to alcohol, parent-child relationship quality, parental involvement, and communication [20,26]. However, studies show that parents do not always act in accordance with evidence-based parenting strategies, which may be related to lack of clear guidance or confidence [27,28]. Although parents actively seek information about illicit drugs and parenting practices to prevent AOD use, primarily from friends or the internet, they often report low-to-moderate confidence in communicating and influencing their children’s choices regarding AOD use [29]. Programs aimed at modifying parenting practices and promoting the use of effective prevention strategies have shown promise in reducing adolescent AOD use [30-32]. Honest communication between parents and adolescents, established on the basis of positive parent-adolescent relationship, has been associated with reduced alcohol use among adolescents [32-34]. This highlights the importance of engaging parents in efforts to reduce harms associated with adolescent AOD use.

It is critical that effective AOD prevention strategies are implemented consistently and widely to alleviate the considerable burden associated with AOD use. Therefore, teachers, school staff, and parents need to have easy access to evidence-based information, strategies, and programs that equip them to respond most effectively. The *Positive Choices* national AOD prevention initiative was funded by the Australian Government Department of Health to enhance access to and implementation of evidence-based AOD prevention strategies within school communities. The initiative responds to a call from school principals for support to implement evidence-based AOD prevention resources (Australian National Council on Drugs; 2013) and was developed iteratively with experts and end users. School staff, parents, and students provided input and feedback across two phases (for full details refer to the study by Stapinski et al [25]): (1) formative consultation to clarify scope and identify user needs and (2) review and feedback on a beta version of the website [35]. The final website was launched in December 2015 and provides web-based training, support, and access to a centralized database of evidence-based AOD prevention programs, recommendations, and resources [25]. The website emphasizes the importance of implementing resources that are supported by research evidence—only evidence-based resources are listed, and each resource page provides a rating to indicate the strength of the supporting evidence according to the Australian National Health and Medical Research Council’s evidence hierarchy [36]. To facilitate implementation, resources are categorized based on purpose and intended audience in a searchable database, with brief factsheets to guide users about when and how to implement evidence-based prevention strategies. A survey was conducted 8 months after the launch to determine the initial impact of *Positive Choices*. Among teachers who accessed the website, most found it useful and reported that they would continue using it, would recommend it to others, felt more comfortable discussing AOD use prevention following website access, and felt that their students were more engaged with AOD education since using the website [25]. When compared with a general teacher sample, teachers who used *Positive Choices* were more likely to consider the evidence base and effectiveness of AOD education resources before using them in class [25]. Despite highlighting these benefits associated with the use of *Positive Choices*, this initial evaluation did not reveal the specific factors or features that facilitated the dissemination of evidence-based AOD prevention strategies.

### Objectives

In this study, we extended this initial evaluation by conducting a more comprehensive examination of the effectiveness and impact of the *Positive Choices* initiative over a 3-year period, between 2017 and 2019. This is the first evaluation of a web-based health initiative that specifically promotes translation of evidence-based AOD prevention resources to school communities, parents, and youth. To guide this evaluation, we applied the reach, effectiveness, adoption, implementation, and maintenance (RE-AIM) framework, which was developed to facilitate comprehensive and rigorous evaluation of health promotion initiatives, spanning these 5 key dimensions. It has been widely applied to evaluate the implementation and real-world impact across a variety of settings, including educational settings [37-40]. Guided by this framework, this study assessed the impact of the *Positive Choices* initiative in increasing awareness and implementation of evidence-based AOD prevention strategies. The study aimed to evaluate the reach of the *Positive Choices* website, its effectiveness in improving access to and uptake of effective AOD prevention strategies, and its adoption in accessing evidence-based prevention strategies. Furthermore, implementation will be evaluated by users’ capacity to interact with the website, and maintenance will be evaluated by users’ intention to continue using the website for evidence-based strategies. The study also obtained feedback from end users to improve and optimize the *Positive Choices* website.

## Methods

Data were collected between 2017 and 2019, from several sources to evaluate the dimensions aligned with the RE-AIM framework, as described in the following sections.

### Reach

#### Overview

Reach was assessed via the measurement of access and awareness, using 2 data sources. Access was measured using site use analytics and operationalized as follows: How many unique users have accessed *Positive Choices*? Awareness was assessed via community awareness surveys and operationalized as follows: Have you heard of *Positive Choices* before?

#### Site Use Analytics

Google Analytics was used to obtain a detailed analysis of website traffic between January 2017 and March 2021. This included information on the number of unique website users and page views, pages viewed per session, and average time users spent on each page.

#### Community Awareness Survey: School Staff and Parents

A web-based survey was administered in July 2018 to assess the Australian community’s awareness and use of *Positive Choices* resources. Using voluntary response sampling, school staff and parents were recruited via targeted advertisements on *Positive Choices* social media channels or mailing lists. Participants who completed the survey were offered the chance to enter a prize draw to win a laptop. Within this general sample, the proportion of the community that was aware of the website was identified by using a single question: Have you heard of *Positive Choices* before? Information was also collected to ascertain the number of participants who were using sources other than *Positive Choices* and how participants were accessing AOD information*.* The same survey was administered after 6 months (November 2018) and 12 months (May 2019) to determine whether awareness increased following a national social media campaign promoting *Positive Choices.*

### Effectiveness

#### Overview

*Positive Choices* aims to improve access to and uptake of effective AOD prevention strategies. Accordingly, effectiveness was assessed via an evaluation survey completed by *Positive Choices* end users (school staff and parents). It was measured through examination of whether engagement with *Positive Choices* was associated with increased intention to implement evidence-based AOD education and was operationalized as follows: Has the use of *Positive Choices* changed users’ intentions in implementing evidence-based teaching and parenting practices?

#### Evaluation Survey: School Staff and Parents

To capture the effectiveness of *Positive Choices*, two anonymous evaluation surveys were administered: the first between August 2017 and September 2017 and the second between May 2019 and June 2019. Using voluntary random sampling, participants were recruited via the *Positive Choices* mailing list and social media campaigns. Eligible participants were school staff or parents or guardians of children or adolescents, were Australian residents, and had access to the internet and a device to complete the survey. Participants were reimbursed for their time with an Aus $40 (US $28) gift voucher (2017) or the opportunity to enter a prize draw to win a laptop (2019).

Participants were asked to spend time reading and interacting with the *Positive Choices* resources, after which they reported on their intentions to implement evidence-based prevention strategies. Data were collected via a web-based survey platform, Survey Monkey, and responses from the 2 evaluations were collated to provide an overview of the effectiveness spanning from 2017 to 2019.

For school staff, questions assessed whether they intended to (1) implement teaching resources that have been tested in schools and proven to prevent AOD use, (2) communicate with students about the risks and effects associated with AOD use, and (3) correct the misperception that AOD use is *the norm*. For parents, questions assessed whether they intended to (1) encourage open communication with their children about AOD, (2) have explicit conversation with their children about AOD use, (3) correct the misperception that AOD use is common, (4) clearly communicate about their expectations to their children, (5) change their own AOD use to model appropriate behavior, (6) avoid parental supply of alcohol, and (7) closely monitor their children’s whereabouts. School staff and parents responded to these behavioral intention items by selecting whether they (1) already do or have done this, (2) plan to do this after viewing *Positive Choices*, or (3) do not plan to do this in the future.

### Adoption and Implementation

Similar to the effectiveness dimension, the adoption and implementation dimensions were assessed via the web-based evaluation surveys. Adoption was assessed by identifying barriers to and enablers of access and uptake of evidence-based prevention strategies by school staff and parents. A single question assessed which of the following characteristics of web-based AOD prevention resources or information were most valued by school staff: (1) evidence-based information, (2) resources that had been tested in schools and proven to prevent AOD use, (3) engaging website, (4) interactive website, (5) website that is easy to navigate and use, and (6) simple and easy-to-understand language. Similarly, enablers for parents and guardians were identified through the same items with the addition of the following two items that pertained specifically to parents: (1) parental strategies that have been proven to be effective and (2) website with advice from other parents.

Implementation was evaluated using the System Usability Scale (SUS) [41]. It is a standardized instrument used to measure the usability of products, software, apps, and websites. It provides participants with 10 usability-related statements on a 5-point Likert scale, ranging from “strongly disagree” to “strongly agree.” SUS scores range from 0 to 100, with score ≥85 representing *exceptional usability*, score between 50 and 70 representing *good usability*, and score <50 representing *unacceptable usability* [41,42]. As such, participants’ capacity to interact with the website to access and subsequently deliver evidence-based strategies was assessed using the SUS scores.

### Maintenance

This dimension was assessed using the website evaluation surveys, as described previously. For the participating school staff and parents, the question assessed whether users intended to access *Positive Choices* in the future. It was conceptualized as follows: Did participants intend to maintain their use of the *Positive Choices* resources?

### General Impression

To assess school staff and parents’ general feedback about the *Positive Choices* website, the evaluation survey included the following open-text items: (1) Do you have any suggestions for improving the website? and (2) Do you have any final comments about the website?

### Data Analysis

The evaluation surveys were administered via *Survey Monkey*, and the data were exported to Stata (version 14; StataCorp), which was used to generate descriptive data, including mean scores and response frequencies. Data from the 2017 and 2019 evaluation surveys were merged to examine reach, effectiveness, adoption, implementation, and maintenance, using descriptive statistics, separately for school staff and parents. The SUS score was calculated by converting raw individual scores for each question to a number; these scores were summed to obtain the total score, 5 was subtracted from the total score of all odd-numbered questions, and 25 was subtracted from the total score of all even-numbered questions. Then, the total score of the new values was multiplied by 2.5 [43].

Data on community awareness were also collected via *Survey Monkey* and exported to Stata to generate descriptive data. The data were examined separately for school staff and parents at baseline, 6 months, and 12 months.

Inductive analysis was used to assess participants’ feedback about *Positive Choices* [44], using the qualitative data analysis software, NVivo (version 12; QSR International). Feedback from participants was examined by SN, who then developed a coding framework. Using the framework, common themes were identified independently by two coders (SN and TG).

### Ethics Approval

The 2017 survey was approved by the Human Research Ethics Committee, University of New South Wales (project number HC12548), and the 2019 survey was approved by the Human Research Ethics Committee, University of Sydney (project number 2018/873).

## Results

### Sample Characteristics: Evaluation Survey

From 2017 to 2019, a total of 200 participants completed the evaluation surveys, of which 73 (36.5%) participants were school staff and 127 (63.5%) were parents.

#### School Staff

Table 1 shows the demographic characteristics of the school staff who completed the evaluation surveys. The average age of the participants was 40 (SD 10.71) years and ranged between 24 and 63 years. Most participants were women (56/73, 77%), resided in New South Wales (NSW; 29/73, 40%), and were based in major cities (36/73, 49%). Most school staff worked in coeducational, secondary schools (ie, years 7-12; 51/73, 70%), and were employed as teachers (48/73, 66%).

**Table 1:** Demographic characteristics of school staff and parents (evaluation survey).

Characteristics		School staff (n=73)	Parents (n=127)
Age (years), range		24-63	26-63
**Gender, n (%)**			
	Women	56 (77)	108 (85)
	Men	16 (22)	19 (14.9)
	Nonbinary	1 (1)	0 (0)
**State or territory, n (%)**			
	Australian Capital Territory	5 (7)	4 (3.1)
	New South Wales	29 (40)	45 (35.4)
	Queensland	10 (14)	23 (18.1)
	South Australia	6 (8)	6 (4.7)
	Tasmania	4 (5)	2 (1.6)
	Victoria	16 (22)	28 (22)
	Western Australia	3 (4)	15 (11.8)
	Northern Territory	0 (0)	4 (3.1)
**Location of school or residence, n (%)**			
	Major city	36 (49)	78 (61.4)
	Regional	33 (45)	48 (37.8)
	Remote	4 (5)	1 (0.8)
**Year levels taught or children’s year levels^a^, n (%)**			
	Foundation	5 (7)	10 (7.9)
	Year 1	8 (11)	6 (4.7)
	Year 2	8 (11)	5 (3.9)
	Year 3	8 (11)	10 (7.9)
	Year 4	7 (10)	13 (10.2)
	Year 5	9 (12)	11 (8.7)
	Year 6	8 (11)	15 (11.8)
	Year 7	38 (52)	26 (20.5)
	Year 8	37 (51)	29 (22.8)
	Year 9	38 (52)	23 (18.1)
	Year 10	40 (55)	24 (18.9)
	Year 11	39 (53)	35 (27.6)
	Year 12	39 (53)	33 (25.9)
	N/A^b^	18 (25)	N/A
**School type, n (%)**			
	Coeducational	63 (86)	N/A
	Single sex	10 (14)	N/A
**Profession, n (%)**			
	Teacher	48 (66)	N/A
	School counselor chaplain	10 (14)	N/A
	Youth worker	2 (3)	N/A
	Researcher	2 (3)	N/A
	Other	11 (15)	N/A
**Employment status, n (%)**			
	Full time	N/A	52 (40.9)
	Part time or casual	N/A	42 (33.1)
	Home duties (including carer)	N/A	19 (14.9)
	Unemployed	N/A	6 (4.7)
	Unable to work	N/A	2 (1.6)
	Student	N/A	5 (3.9)
	Other	N/A	1 (0.8)

^a^Respondents were able to select multiple responses; thus, the column total for this item does not add to 100%.

^b^N/A: not applicable.

#### Parents

Table 1 also shows the demographic characteristics of the parents who completed the surveys. The average age of the parents was 46 (SD 6.63) years, and they were aged between 26 and 63 years. Most parent respondents were women (108/127, 85%), resided in NSW (45/127, 35.4%), and were based in major cities (78/127, 61.4%). Most parents were employed full time (52/127, 40.9%), with most of them having children who attended secondary school (ie, years 7-12; 122/127, 96.1%).

### Sample Characteristics: Community Awareness Survey

#### Overview

A total of 1435 participants completed the community awareness surveys across baseline, 6-month follow-up, and 12-month follow-up. At baseline, participants were 48.55% (201/414) school staff and 51.45% (213/414) parents. At the 6-month follow-up, participants were 51.45% (249/484) school staff and 48.55% (235/484) parents. Finally, at the 12-month follow-up, participants were 50.09% (269/537) school staff and 49.91 (268/537) parents.

#### School Staff

All school staff who completed the community awareness surveys were high school staff (719/719, 100%), most resided in NSW (259/719, 36%), and were based in major cities (381/719, 52.9%; Table 2). Most school staff (432/719, 60.1%) worked in public schools in a number of different roles (eg, head teacher, curriculum coordinator, language teacher, mathematics or science teacher, advanced skills teacher, student support or school counselor, and teacher assistant) and worked with Aboriginal or Torres Strait Islander students.

**Table 2:** Demographic characteristics of school staff and parents (community awareness survey).

Characteristics		School staff (n=719), n (%)	Parents (n=716), n (%)
**State or territory**			
	Australian Capital Territory	5 (0.7)	15 (2.1)
	New South Wales	259 (36)	207 (28.9)
	Queensland	147 (20.4)	136 (18.9)
	South Australia	46 (6.4)	75 (10.5)
	Tasmania	32 (4.5)	19 (2.7)
	Victoria	134 (18.6)	168 (23.5)
	Western Australia	85 (11.8)	88 (12.3)
	Northern Territory	11 (1.5)	8 (1.1)
**Location of school or residence**			
	Major city	381 (53)	379 (52.9)
	Regional	185 (25.7)	192 (26.8)
	Rural	93 (12.9)	93 (12.9)
	Remote	34 (4.7)	29 (4.1)
	Very remote	26 (3.6)	23 (3.2)
**Works with Aboriginal and/or Torres Strait Islander student (school staff) or identified as Aboriginal and/or Torres Strait Islander (parents)**			
	Yes	568 (78.9)	21 (2.9)
	No	122 (16.9)	687 (95.9)
	Prefer not to answer	29 (4)	8 (1.1)
**School**			
	Public	432 (60.1)	N/A^a^
	Faith-based	145 (20.2)	N/A
	Independent	95 (13.2)	N/A
	Coeducational	21 (2.9)	N/A
	Single sex (female)	12 (1.7)	N/A
	Single sex (male)	9 (1.3)	N/A
	Selective	5 (0.7)	N/A
**Family structure^b^**			
	Single-parent household	N/A	192 (26.8)
	2-parent household	N/A	500 (69.8)
	1 child	N/A	69 (9.6)
	2-4 children	N/A	397 (55.4)
	≥5 children	N/A	39 (5.4)
	Step siblings (cohabiting)	N/A	27 (3.8)
	Prefer not to answer	N/A	2 (0.3)

^a^N/A: not applicable.

^b^Respondents were able to select multiple responses; thus, the column total for this item does not add to 100%.

#### Parents

All parents who completed the community awareness surveys (716/716, 100%) were parents of high school students. Most of them resided in NSW (207/716, 28.9%), were based in major cities (379/716, 52.9%), and did not identify as Aboriginal and/or Torres Strait Islander (687/716, 95.9%; Table 2). Most parents were part of 2-parent households (500/716, 69.8%) and had between 2 and 4 children (397/716, 55.4%).

### Evaluation of the Positive Choices Website (RE-AIM Framework)

#### Reach: How Many Users Have Accessed Positive Choices?

##### Overview

**Figure 1:**
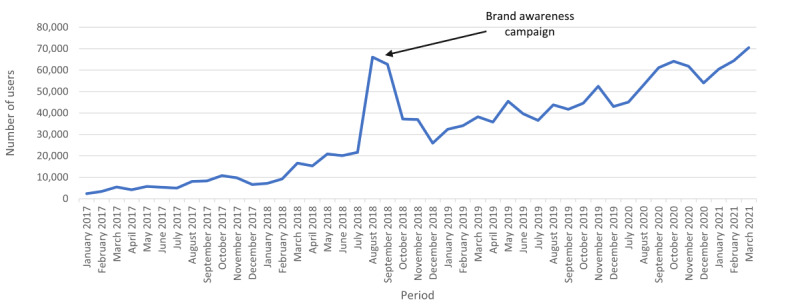
Monthly Positive Choices website visitors, between January 2017 and March 2021.

Between January 1, 2017, and March 31, 2021, *Positive Choices* had been visited by 1.7 million unique users, of which 1.53 million (90%) were first-time users. Figure 1 shows that the number of monthly users has consistently increased since January 2017. Similarly, the number of monthly page views have increased from 8367 in January 2017 to 123,699 in March 2021. In terms of geographical reach, most of the website’s visitors are Australian residents (41%); however, the website is also frequently accessed by international users from countries such as the United States, India, the Philippines, and the United Kingdom.

##### Community Awareness

The spike in website visitors seen between July 2018 and November 2018 (Figure 1) coincided with the community awareness campaign run from June 2018 to October 2018. During this period, there was an 84% increase in the average monthly users of the *Positive Choices* website from 20,115 to 37,047. There was also an increase in users’ engagement on the *Positive Choices* Facebook page, with the number of *likes* increasing by 86%, from 670 to 1243. Results from the surveys showed that awareness about *Positive Choices* increased among school staff from 8% at baseline to 14% at 6 months (immediately after the awareness campaign) and 15% at 12 months. Among parents, awareness increased from 15% at baseline to 22% at 6 months (immediately after the awareness campaign) before returning to 15% at 12 months.

According to the 12-month survey, of the school staff who were not using *Positive Choices* for AOD education resources and information (191/269,71%), 45.5% (87/191) were using Alcohol and Drug Foundation, 18.3% (35/191) were using Drug Free Australia, 14.1% (27/191) were using Australian Drug Information Network, 18.3% (35/191) were using Drug Help, 4.7% (9/191) were using Prevention Smart, and 6.3% (12/191) were using other sources (*headspace*, general practitioners, guidance officer, etc). Similarly, among the same group that was not using *Positive Choices,* approximately 69.6% (133/191) of school staff used websites (government, Google, or other websites) to access AOD information; 43.5% (83/191) obtained information through school administrators (eg, Department of Education and Catholic Education Office); and 13.6% (26/191) used other sources including AOD professionals, books, police, and so on. Similarly, among parents who were not using *Positive Choices* (201/268, 75%), 60.7% (122/201) parents reported that they accessed information through Google or other websites; 26.4% (53/201) from other parents; 30.3% (61/201) through government websites; 8.5% (17/201) through their general practitioners; 4.9% (10/201) through their children’s classroom teachers; and 7.9% (16/201) of parents were using other sources such as *headspace*, other health professionals, presenters at local AOD prevention events, and so on.

#### Effectiveness: Has the Use of Positive Choices Changed Users’ Teaching and Parenting Practices?

##### School Staff

Table 3 shows that approximately half of the school staff sample (35/73, 48%) were already implementing AOD education resources that were tested and found to be effective in schools; among those who were not implementing those resources, 89% (34/38) intended to after using *Positive Choices*. Most respondents were already communicating to their students about the risks and effects of AOD use (52/73, 71%), and of the remaining respondents, 90% (19/21) intended to do so after using *Positive Choices*. When it came to correcting students’ misperceptions about AOD use, most respondents were already doing this (52/73, 71%) and of the remaining respondents, 81% (17/21) intended to commence this after viewing the *Positive Choices* website.

**Table 3:** Effectiveness in changing intentions to use evidence-based strategies (school staff).

	Respondents who are currently implementing evidence-based strategy (n=73), n (%)	Among respondents who were not implementing evidence-based strategies, those who intend to after viewing *Positive Choices*, n (%)	Respondents who will not implement evidence-based strategies in the future, n (%)
Implement teaching resources that were tested in schools and proven to prevent alcohol and drug use	35 (48)	34 (89)^a^	4 (11)^a^
Communicate with students about the risks and effects of alcohol and drug use	53 (73)	18 (90)^b^	2 (10)^b^
Correct the misperception that alcohol and other drug use is common or “the norm”	52 (71)	17 (81)^c^	4 (19)^c^

^a^Sample size, n=38.

^b^Sample size, n=20.

^c^Sample size, n=21.

##### Parents

Table 4 shows that most parents reported that they were already having open (100/127, 78.7%) and clear conversations (95/127, 74.8%) with their children about AOD use. Among those parents who were not already doing this, 85% (23/27) planned to have open and 78% (25/32) planned to have clear conversations with their children after viewing resources on *Positive Choices*. Most parents reported that they were already having explicit conversations about AOD with their children (88/127, 69.3%), and most of the remaining parents (36/39, 92%) planned to do so after using *Positive Choices*. Most parents in the survey were already avoiding supplying alcohol to their children (103/127, 81.1%). Of those who were not doing this, 38% (9/24) reported that they would avoid supplying alcohol after viewing resources on *Positive Choices*. Similar to the school staff, most parents reported that they were already correcting misperceptions about AOD use being *the norm* (80/127, 62.9%), and of the remaining parents, 83% (39/47) planned to implement this behavior after using *Positive Choices*. Most parents reported that they have already adapted their own AOD use to model appropriate behavior for their children (87/127, 68.5%), and of the remaining parents, 45% (18/40) planned to modify their behavior after using *Positive Choices*. After viewing resources on *Positive Choices*, most parents reported that they will monitor their children’s whereabouts more closely (64/74, 86%).

**Table 4:** Effectiveness in changing intentions (parents).

	Respondents who are currently implementing evidence-based strategy (n=127), n (%)	Among respondents who were not implementing evidence-based strategies, those who intend to after viewing *Positive Choices*, n (%)	Respondents who will not implement evidence-based strategies in the future, n (%)
Encourage open communication with my child about alcohol and other drugs	100 (78.7)	23 (85)^a^	4 (15)^a^
Have an explicit conversation with my child about alcohol and other drugs	88 (69.3)	36 (92)^b^	3 (8)^b^
Correct the misperception that alcohol and other drug use is common or “the norm”	81 (63.8)	38 (83)^c^	8 (17)^c^
Clearly communicate my expectations about drug and alcohol use to my child	95 (74.8)	25 (78)^d^	7 (22)^d^
Change my own drug or alcohol use to model appropriate behavior	87 (68.5)	18 (45)^e^	22 (55)^e^
Avoid supplying my child with alcohol	103 (81.1)	9 (38)^f^	15 (63)^f^
Monitor my child’s whereabouts more closely	54 (42.5)	63 (86)^g^	10 (14)^g^

^a^Sample size, n=27.

^b^Sample size, n=39.

^c^Sample size, n=46.

^d^Sample size, n=32.

^e^Sample size, n=40.

^f^Sample size, n=24.

^g^Sample size, n=73.

#### Adoption and Implementation

##### School Staff

###### Overview

School staff reported that they spent between 5 and 10 hours per semester on AOD education. Most school staff rated the following factors highly (either “very important” or “important”) when selecting web-based AOD prevention resources: evidence-based information (69/73, 95%), easy-to-navigate and easy-to-use website (69/73, 95%), engaging website (70/73, 96%), simple and easy-to-use language (68/73, 93%), and resources tested in school and found to be effective (62/73, 85%). Although school staff also valued interactive features of prevention websites (55/73, 75%), the proportion of participants who rated this factor highly was lower than those for the other factors facilitating effective AOD prevention. When discussing AOD topics, school staff displayed high levels of confidence, with 41% (30/73) feeling “very confident,” 38% (28/73) feeling “confident,” and 16% (12/73) feeling “somewhat confident”; only 4% (3/73) reported feeling “not very confident.”

###### Implementation

Regarding usability of the *Positive Choices* website, the mean SUS score was 75 (SD 15.1; range 35-100), indicating *good* website usability.

##### Parents

###### Overview

Most parents rated the following factors highly (either “very important” or “important”) when selecting web-based AOD prevention resources: evidence-based information (119/127, 93.7%); strategies that were tested and proven to be effective in AOD use prevention (119/127, 93.7%); simple and easy-to-use language (109/127, 85.8%); and engaging (115/127, 90.6%), interactive (72/127, 56.7%), and easy-to-navigate and easy-to-use (123/127, 96.9%) website. In addition, 57.5% (73/127) of the parents valued prevention advice from other parents.

Parents also reported high confidence in discussing AOD topics: 48.8% (62/127) were “very confident,” 31.5% (40/127) were “confident,” 17.3% (22/127) felt “somewhat confident,” and 2.4% (3/127) felt “not very confident.” These ratings suggest that confidence was not a significant barrier to evidence-based prevention in this sample. This is in contrast to the general community sample, from the community awareness survey, where lack of confidence was reported as the greatest barrier for parents to having conversations with their children about AOD use.

###### Implementation

The mean SUS score for the parent group was 74 (SD 12.9; range 40-100), indicating *good* website usability, similar to that found in the school staff group.

#### Maintenance: Are Users Likely to Continue Accessing Positive Choices?

##### School Staff

Most school staff reported that they would use *Positive Choices* in the future (66/73, 90%), use the website frequently (53/73, 73%), and recommend the website to friends or colleagues (68/73, 93%).

##### Parents

Most parents also reported that they would use *Positive Choices* in the future (116/127, 91.3%), use the website frequently (78/127, 61.4%), and recommend the website to friends or colleagues (113/127, 88.9%).

#### User Feedback on the Website

The main themes that emerged from the analysis of users’ feedback on the website centered around the website’s content and features and promotion and increasing the usability and diversity of the website. Most responses from participants were suggestions to improve the *Positive Choices* website’s layout to make it less cluttered and more visually appealing and engaging (especially to the student user group) and to improve website navigation. The other subthemes that were identified included feedback about making the website more accessible to multicultural communities and students with additional learning needs, suggestions for new content and features, and the need to increase promotion. In response to this feedback, redesign of the *Positive Choices* website was conducted from December 2020 to January 2021, in consultation with a specialist user experience company. The themes and subthemes from the participants’ feedback and actions taken in response to user suggestions are described in Multimedia Appendix 1.

## Discussion

### Principal Findings

Adolescence is a time of increased susceptibility to engaging in risk-taking behaviors such as AOD use. Prevention strategies designed to target modifiable risk factors have been demonstrated to be effective in reducing AOD use and related harms among adolescents [16,18]. Despite this evidence, effective AOD prevention strategies are not widely implemented in schools or by parents. *Positive Choices* was developed to help overcome some of the barriers faced by school communities and parents and promote widespread implementation of evidence-based prevention. This study used the RE-AIM framework to evaluate the success of *Positive Choices* in increasing awareness and implementation of evidence-based AOD prevention practices among school staff and parents across the five domains: reach, efficacy, adoption, implementation, and maintenance.

A total of 5 years following its launch, this Australian AOD prevention website has reached >1.7 million users, and the page views have continued to grow. The user reach of *Positive Choices* has expanded beyond Australia, with 59% of users currently being international. Promotion via a national social media campaign proved to be an effective method for increasing awareness within the Australian community, with awareness increasing from 8% to 14% among school staff and from 15% to 22% among parents after the campaign. The findings suggest that sustained promotion efforts may be required to maintain high levels of awareness about *Positive Choices*. Even among participants who were not using *Positive Choices*, most reported using other web-based sources to obtain AOD education information and resources, thus supporting the value of the internet as a tool for disseminating health information and promoting evidence-based prevention strategies [45,46].

Although evidence shows that effective AOD prevention strategies are not commonly implemented by schools [24] or parents [27], most school staff and parents in this study reported that they were already implementing evidence-based AOD prevention strategies. Moreover, following a brief interaction with the website, most school staff and parents reported an intention to shift toward evidence-based practices in cases where they were not already following the recommended prevention strategies for young people. The exception was the recommendation that parents modify their own alcohol use to model appropriate behavior for their children, which may indicate that changing their own behavior is a significant challenge for parents, which requires additional attention and support. The sample intended to maintain their use of the *Positive Choices* website in the future to obtain evidence-based information and resources. The enablers that facilitated adoption and implementation were ease of availability and good website usability. Both user groups reported high level of confidence in communicating about AOD-related topics. Study participants’ suggestions for improvement pertained to some of these enablers (eg, improving navigation and making the website more engaging), and thus, have informed a recent website update.

The widespread use of web-based health promotion tools and websites by school staff and parents to access information and resources highlights the need for quality control measures. This will ensure that they are evidence-based, up to date, and engaging and use simple language. In addition, the websites themselves should be accessible and easy to use and navigate. Findings from robust evaluations will allow critical assessment of the benefits of such tools and websites, inform content, and inform website updates and developments to optimize their usability. Although there have been evaluations of mental health information websites [47,48], to the best of our knowledge, this is the first evaluation of a web-based health initiative that specifically promotes the translation of evidence-based AOD prevention resources for school communities, parents, and youth.

### Limitations

A limitation of the study is that the findings rely on users’ self-reported intentions to implement evidence-based strategies, rather than actual assessment of their subsequent behavior. Furthermore, the study design assessed participants’ feedback and behavioral intentions after they interacted with the website for a relatively short period. Future studies with a pre-post study design will enable a more comprehensive evaluation, including assessment of whether the website affected subsequent behaviors and implementation of evidence-based strategies by school staff and parents. Another limitation is that the targeted campaign used to recruit participants for the evaluation study may have resulted in a sample selection bias. Most of the surveyed school staff and parents in the current sample reported that they were already implementing evidence-based prevention strategies and were confident about discussing alcohol and drug use. These results contrast with previous evidence suggesting low confidence and implementation of evidence-based AOD prevention strategies among parents and in schools [24,27], and thus, may reflect that our recruitment strategies attracted a sample who were already interested in and aware of evidence-based AOD prevention approaches.

### Conclusions

The findings from the evaluation of *Positive Choices* demonstrate that the website reached 1.7 million users, and it has the capacity to be an effective strategy for disseminating evidence-based AOD prevention information and resources. Furthermore, this evaluation highlights the importance of investing in ongoing promotion to maintain or enhance awareness of health websites. As the use and acceptability of health education websites increase, developers and health care and research teams should ensure that health websites developed in the future are easy to navigate, are engaging, use simple language, contain evidence-informed resources, and are supported by ongoing promotional activities. This study provides methodology and recommendations to guide future evaluations of web-based health tools to determine their effect on behavioral and health outcomes.

## Appendix

Multimedia Appendix 1 Themes and subthemes from participants’ feedback on the website.

## Abbreviations

AOD: alcohol and other drug
NSW: New South Wales
RE-AIM: reach, effectiveness, adoption, implementation, and maintenance
SUS: System Usability Scale
