# Supplementary material for: Evaluation of Positive Choices, a National Initiative to Disseminate Evidence-Based Alcohol and Other Drug Prevention Strategies: Web-Based Survey Study
Source: JMIR Pediatr Parent. 2022 Aug 26;5(3):e34721. doi: 10.2196/34721 (PMC9463616; doi:10.2196/34721)
Supplement: Multimedia Appendix 1 [file pediatrics_v5i3e34721_app1.docx]

**Multimedia Appendix 1: Themes and subthemes from participants’ feedback on the website**

| **Themes** | **Subthemes** | **School staff comments** | **Parent comments** | **Actions taken** |
| --- | --- | --- | --- | --- |
| **User friendly website** | Use of minimalist approach /Make pages less busy | “A lot of information blends into each other on the mobile device, for example in search results. Maybe adopt a minimalist approach to search results or incorporate stronger use of fonts, colours to differentiate.” | “The website banner was a bit cluttered... I like things easy on the eye and to have my attention drawn to the important areas. When something is cluttered, this isn't as effective.” | The designs of the homepage and user landing pages have been updated to improve the overall layout, navigation, search function, and accessibility. |
|  |  | “There is a lot of information in the portal, which is a good thing, but it could possibly be broken up in different ways so not all content is provided in chunks of text.” | “Reduce the verbosity of the prose. Use dot points effectively to allow parents with limited time or lower literacy and easier point of access.” |  |
|  | Visually appealing and engaging | “Organise the way the resources are displayed, make it more engaging.” | “Less stimuli visually.” |  |
|  |  | “Make the start screen more user friendly to get parents, teachers and students to different parts of the webpages.” | “Try to make the graphics more relevant. Be less corporate in your presentation.” |  |
|  | Improve search feature | “Overall it was engaging and attractive but a little hard to navigate.” | “It would be great if search function returned a “close” result in cases of spelling mistakes.” |  |
| **Diverse populations** | Multicultural communities | “More multicultural pictures. Different tabs for language links.” | “Perhaps make an easier design and integrate other languages.” | Images throughout the website have been updated to reflect multicultural Australian society. |
|  |  | “[Add] multilingual materials and access.” | “Additional languages and a button to turn reading into audio for people who can't read.” | Resources are being developed in consultation with multicultural health workers and communities. These resources will be available in three languages i.e., Arabic, Hindi, and Mandarin. |
|  | Students with disabilities or additional learning needs | “Link availability, resources for students in support classes (intellectual disability) would be useful.” |  | A website accessibility audit was conducted to ensure that the website meets Web Content Accessibility Guidelines 2.1 AA, and is correctly designed and coded for the use of people with disabilities. Additionally, a text to speech and text translate functionality was added to the website to make the website accessible for visually impaired users and those who speak languages other than English. |
| **Website content and features** | Suggestions for content | “More realistic with pictures and stats.” | “Personal stories and not all horror stories because teens just don't respond to scare tactics. Sharing experiences.” | Quotes from users have been added on various pages. Story writing competition planned, the winning story will be shared on the website. |
|  |  |  | “Use more realistic/down to earth photographs.” | New images added throughout the website, they are realistic and reflects diversity. |
|  |  |  | “Maybe organise an online function for people who experience difficulty using the site.” | Webinar to introduce users to the new Positive Choices website, resources available, and to demonstrate how users could find drug education resources on it. |
|  | Suggestion for new features | “Links to current news stories.”  “Include current news articles that students and staff could use.” | “Maybe incorporate some of the interactive games.” | Addition of a new blog section through which latest research and relevant news stories are shared. |
|  |  | “Maybe a feature where teachers can chat, or a forum to share ideas.” | “Just provide links to further reading or have a bibliography for where you got the statistics.” | Addition of a drop-down bibliography. |
| **Promotion** | Need for more promotion to make it more widely known | “I think it is a brilliant resource and will be making more teachers that I work with aware of it, as I am not sure that this resource is widely known about.” | “Market it to parents and other influential adults of young people from 10 years upwards.” | Recent increase in promotions targeted at school staff and parents. |
|  |  | “It is a valuable and useful resource - I think there should be greater promotion about it.” | “It's great. Never heard of it before.” |  |
